# Quantifying heterogeneity in individual participant data meta-analysis with binary outcomes

**DOI:** 10.1186/s13643-017-0630-4

**Published:** 2017-12-06

**Authors:** Bo Chen, Andrea Benedetti

**Affiliations:** 1 Department of Epidemiology, Biostatistics and Occupational Health, McGill University, Purvis Hall, 1020 Pine Avenue West, Montreal, Canada; 2 Respiratory Epidemiology and Clinical Research Unit, McGill University, 2155 Guy St. 4th Floor, Office 412, Montreal, 24105 Canada

**Keywords:** Individual participant data meta-analysis (IPD-MA), Heterogeneity, Two-stage and one-stage approaches, *I*^2^

## Abstract

**Background:**

In meta-analyses (MA), effect estimates that are pooled together will often be heterogeneous. Determining how substantial heterogeneity is is an important aspect of MA.

**Method:**

We consider how best to quantify heterogeneity in the context of individual participant data meta-analysis (IPD-MA) of binary data. Both two- and one-stage approaches are evaluated via simulation study. We consider conventional *I* ^2^ and *R* ^2^ statistics estimated via a two-stage approach and *R* ^2^ estimated via a one-stage approach. We propose a simulation-based intraclass correlation coefficient (ICC) adapted from Goldstein et al. to estimate the *I* ^2^, from the one-stage approach.

**Results:**

Results show that when there is no effect modification, the estimated *I* ^2^ from the two-stage model is underestimated, while in the one-stage model, it is overestimated. In the presence of effect modification, the estimated *I* ^2^ from the one-stage model has better performance than that from the two-stage model when the prevalence of the outcome is high. The *I* ^2^ from the two-stage model is less sensitive to the strength of effect modification when the number of studies is large and prevalence is low.

**Conclusions:**

The simulation-based *I* ^2^ based on a one-stage approach has better performance than the conventional *I* ^2^ based on a two-stage approach when there is strong effect modification with high prevalence.

**Electronic supplementary material:**

The online version of this article (doi:10.1186/s13643-017-0630-4) contains supplementary material, which is available to authorized users.

## Background

Meta-analysis (MA) is a statistical method used to draw an overall conclusion based on the total evidence by reviewing previous research work systematically and pooling effect estimates together [1]. MA is an important tool, widely used, and applied in evidence-based medicine [2].

Individual participant data meta-analyses (IPD-MA), collect line by line participant data from each included study, rather than estimates of the parameter of interest. IPD-MA offer several advantages over aggregate data MA (AD-MA) and are considered the gold standard in meta-analytic techniques [3].

Heterogeneity of effect estimates is an important consideration in both AD-MA and IPD-MA. Heterogeneity exists if the true effects vary across studies more than would be expected by chance alone. The estimated inter-study variance (*τ* ^2^) of the parameter of interest is the most direct measure of heterogeneity, but interpretation, particularly deciding what might be a problematic level of heterogeneity, is difficult, despite some practical suggestions [4]. The *I* ^2^, originally proposed by Higgins and Thompson, meets three important criteria for any measure of heterogeneity: it monotonically increases with between-study variance; it is not varied by changing the scale; and it is not affected by the number of studies [5]. Importantly, despite some limitations [4], the *I* ^2^ remains the most often reported measure of heterogeneity and is easily interpretable, appealing to clinicians.

There are two approaches to analyze the data from IPD-MA: the two-stage approach and the one-stage approach. In the two-stage approach, each study is analyzed separately, then standard meta-analytic techniques are applied, and heterogeneity may be quantified by usual methods. Alternatively, in the one-stage approach, a mixed model is fit and the data is analyzed altogether, accounting for the correlation that may exist between subjects in the same study and allowing the estimated effect to vary across studies. A review of statistical methods used in IPD-MA of binary outcomes found that most do not report any measure of heterogeneity [6]. While some measures of heterogeneity are easily obtained from a one-stage model, the *I* ^2^ is not. Our objective in this work was to consider various approaches to quantifying heterogeneity in IPD-MA of binary outcomes analyzed via the one-stage approach. We propose a method to obtain an *I* ^2^ from a one-stage model and evaluate it and other possible measures via simulation study.

### Metrics of heterogeneity in IPD-MA with binary outcomes

In this section, we describe various measures of heterogeneity that may be used. Here, we consider that the primary analysis is a one-stage analysis of IPD-MA of dichotomous outcome data. Below, we describe four possible measures of heterogeneity: (1) the conventional *I* ^2^ from the corresponding two-stage analysis; (2) the *R* from the corresponding two-stage analysis; (3) a new metric: the *I* ^2^ from the one-stage approach; and (4) the *R* from the one-stage approach.

#### Between-study variance (*τ*^2^)

The between-study variance, *τ* ^2^, quantifies the heterogeneity in IPD-MA directly. A large value of $\hat {\tau }^{2}$ indicates that heterogeneity exists among the studies. However, the *τ* ^2^ is not ideal, since interpretation is difficult: there is no standard criteria to determine the level of heterogeneity (low, moderate, substantial), because the range is from 0 to *∞* [5, 7]. All other approaches to quantify heterogeneity rely on *τ* ^2^.

We might estimate the *τ* ^2^ via the two-stage or the one-stage approach. For the two-stage approach, we estimate $\tau ^{2}_{\mathrm {two-stage}}$ via the method described by DerSimonian, Laird, and Whitehead [7–9]:

1 $$ \hat{\tau}^{2}=\max\left(\frac{Q-(N-1)}{\sum_{i=1}^{N}\hat{\omega}_{i}-\frac{\sum_{i=1}^{N}\hat{\omega}_{i}^{2}}{\sum_{i=1}^{N}\hat{\omega}_{i}}},0\right)  $$

where *N* is the number studies, $\hat {\omega _{i}}$ is the reciprocal of estimated within-study variance, and *Q* represents Cochran’s heterogeneity statistic [5, 10, 11].

A two-stage analysis proceeds as follows. Consider a MA of a binary outcome in *N* studies. In the first stage, we fit the logistic regressions in each of the *N* studies:

$$y_{j}\sim \text{Bernoulli}(p_{j}) $$

2 $$ \text{logit}(p_{j})=\beta_{0}+\beta_{1}x_{j}  $$

where *p*_*j*_ is the true response probability for the positive result of the *j*th individual in this study, *β*_0_ represents the intercept, and *x*_*j*_ indicates their treatment status. This model could be expanded to include effect modifiers.

In the first stage, we obtain $\hat {\beta }_{1i}$ the estimated log odds ratio in study *i* for *i*=1,2,..,*N* [12], and the variance of the estimated log odds ratio ($\text {var}(\hat {\beta }_{1i})$) for each one of the *N* studies.

In the second stage, we consider:

3 $$ \hat{\beta}_{1i}\sim \text{Normal}\left(\beta_{1},\tau^{2}_{\beta_{1}}+\text{var}(\hat{\beta}_{1i})\right)  $$

where $\tau ^{2}_{\beta _{1}}$ ($\tau ^{2}_{\mathrm {two-stage}}$) represents the respective degree of heterogeneity between studies [12]. Here, we assume the covariance between the parameter estimates (*β*_0*i*_ and *β*_1*i*_) are equal to 0, which means that we pool the treatment-outcome associations (*β*_1*i*_) together [12]. This is similar to the classic DerSimonian and Laird random-effects model [8, 13] and allows us to obtain an estimate of the between-study variance $\tau ^{2}_{\mathrm {two-stage}}$ [12], as in Eq. 1.

For the one-stage approach, with binary data, we may estimate the $\tau ^{2}_{\mathrm {one-stage}}$ from a generalized linear mixed model (GLMM) [14–16]. Under the one-stage random-effects model, for each study, a study-specific intercept and treatment effect may be estimated. The study-specific intercept and treatment effects are assumed to come from a bivariate normal distribution [3, 17, 18]. Considering again a MA of a binary outcome in *N* studies:

$$y_{ij}\sim \text{Bernoulli}(p_{ij}) $$

4 $$ \text{logit}(p_{ij})= (\beta_{0}+\mu_{0i})+(\beta_{1}+\mu_{1i})x_{ij}  $$

5 $$ \left[\begin{array}{l} \mu_{0i} \\ \mu_{1i} \end{array}\right] \sim \text{MVN}\left(\left[\begin{array}{l} 0 \\ 0 \end{array}\right], \left[\begin{array}{ll} \tau^{2}_{0}&\rho\tau_{0}\tau_{1} \\ \rho\tau_{0}\tau_{1}&\tau^{2}_{1} \end{array}\right]\right)  $$

where *p*_*ij*_ represents the true response probability for the positive result of the *j*th individual in the *i*th study and *x*_*ij*_ indicates their treatment status. *β*_1_ is the parameter of interest, which represents the pooled log odds ratio, and $\tau ^{2}_{1}$ is the between-study variance ($\tau ^{2}_{\mathrm {one-stage}}$).

#### *I*^2^ statistic

Using a two-stage approach, consider a MA of *N* studies for the parameter of interest, called *θ*. Under the assumption that the estimated sampling variances are known and equal for all studies ($\sigma ^{2}_{1}=\sigma ^{2}_{2}=\sigma ^{2}_{3}=...=\sigma ^{2}_{N}=\sigma ^{2}=1/\omega _{i}$), Higgins and Thompson [5] defined a measurement function *I* ^2^ for quantifying the unexplained heterogeneity, where $E(\theta _{i})=\theta, V(\theta _{i})=\tau ^{2}, E(\hat {\theta }_{i}|\theta _{i})=\theta _{i}$, and $V(\hat {\theta }_{i}|\theta _{i})=\sigma ^{2}$.

They proposed to estimate $I^{2}_{\mathrm {two-stage}}$ as [5]:

6 $$ \hat{I}^{2}=\frac{\hat{\tau}^{2}}{\hat{\tau}^{2}+\hat{\sigma}^{2}}  $$

where

7 $$ \hat{\sigma}^{2}=\frac{N-1}{\sum_{i=1}^{N}\hat{\omega}_{i}-\frac{\sum_{i=1}^{N}\hat{\omega}_{i}^{2}}{\sum_{i=1}^{N}\hat{\omega}_{i}}}.  $$

While the *I* ^2^ is usually presented as a percentage varying from 0 to 100%, we present it as a proportion varying from 0 to 1.

In clustered data analyses, the *I* ^2^ is very similar to the intraclass correlation coefficient (ICC) [5, 19]. The ICC is the ratio of the between-cluster variance to the total variance in the outcome [20]. It provides a quantitative measure of the amount of heterogeneity across clusters [14]. With binary data, estimating the ICC from a GLMM is possible, though more complicated [14–16]. Several measures have been proposed as estimators of the ICC for binary data [14, 21, 22], though none have been evaluated as measures of inter-study heterogeneity for IPD-MA. Goldstein et al. proposed a simulation-based approach that relies on partitioning the variation in the multilevel model to estimate an ICC for binary outcomes [22]. We propose to adapt this ICC estimator to estimate the *I* ^2^ in a one-stage IPD-MA. The algorithm is as follows:

- **Step 1** Fit a random effects model to the data by using a GLMM, and adjust for possible effect modifiers if desired.
- **Step 2** Simulate a large number (e.g. *m*=5000) of values from a normal distribution (e.g. Eq. 5), using the estimated covariance matrix from the multilevel random effect logistic regression fitted in Step 1. We denote these as *μ*_0,*ij*_,*μ*_1,*ij*_.
- **Step 3** Using the fitted model from Step 1, we estimate the log odds ratio for each subject ($\nu _{1,ij}=\hat {\beta }_{1}+\mu _{1,ij}$) in the dataset, and then estimate the variance as *ν*_1_=*V*(*ν*_1,*ij*_).
- **Step 4** Estimate *p*_*ij*_ by using the fitted model (from Step 1) and simulated random effect values (from Step 2).
  - replacing (*μ*_0*i*_, *μ*_1*i*_) by (*μ*_0,*ij*_,*μ*_1,*ij*_) for each subject
  - plugging in the fixed effect estimator from the fitted model and the covariates from the dataset
  - taking the inverse logit, we obtain the $\hat {p}_{ij}$ for each individual
- **Step 5** Using the results from Step 4, it is easy to deduce the variance of the estimated log odds ratio via the Delta method, $\nu _{2,ij}=\frac {1}{n\hat {p}_{ij}(1-\hat {p}_{ij})}$, where *n* is the average number of subjects among all of the studies. Finally, we obtain *ν*_2_=*E*(*ν*_2,*ij*_).
- **Step 6** The $I^{2}_{\text {one-stage}}$ is now estimated as

8 $$ \hat{I}^{2}=\frac{\nu_{1}}{\nu_{1}+\nu_{2}}.  $$

#### *R* statistic

The *R* statistic is the square root of the ratio of the variance of the summary statistic from the random-effects model divided by the variance of the summary statistic from the fixed-effects model. It quantifies the inflation of the confidence interval in the presence of inter-study heterogeneity [5]. If the estimated value of *R* is close to 1, then inference from the random- and fixed-effects models are similar [5]. However, unexplained between-study heterogeneity may exist when the estimate of *R* is greater than 1. Interpretation of *R* is difficult, for the same reasons as for *τ* ^2^.

For the two-stage approach, we may estimate *R*_two−stage_ as

9 $$ \hat{R}=\frac{se(\hat{\beta}_{1R})}{se(\hat{\beta}_{1F})}  $$

where $se(\hat {\beta }_{1F})$ is the standard error of the estimated pooled log odds ratio in the fixed-effects model and $se(\hat {\beta }_{1R})$ is the standard error of the estimated pooled log odds ratio in the random-effects model (Eq. 3).

For the one-stage approach, we may fit a GLMM with random intercept and fixed slope. The standard error of the estimated *β*_1_ (pooled log odds ratio) from that model with fixed intercept may be denoted as $se(\hat {\beta }_{1F})$. We denote the standard error of the estimated *β*_1_ from model 4 as $se(\hat {\beta }_{1R})$. The estimated *R*_one−stage_ from a one-stage model may then be estimated using Eq. 9.

## Methods

We use simulations to investigate the performance of (i) conventional *I* ^2^ and *R* ^2^ based on a two-stage approach and (ii) simulation-based *I* ^2^ and *R* ^2^ based on a one-stage approach. We generated datasets that consisted of three variables: the binary treatment status, a binary effect modifier, and a binary outcome. Each combination of data generation parameters was used to generate 1000 datasets. We considered 84 distinct data generation scenarios (see Table 1).

**Table 1:** Parameter values for generating datasets

Parameter	Value
The number of studies (*N*)	15, 30
Prevalence (*p*_pre_)	0.3, 0.7
True between-study variance (*τ* ^2^)	0.5, 1, 1.5
No effect modification (*β*_*w*_, *β*_*xw*_)	(0, 0)
With weak effect modification (*β*_*w*_, *β*_*xw*_)	(1, 1)
With moderate effect modification (*β*_*w*_, *β*_*xw*_)	(1, 3)
With moderate effect modification (*β*_*w*_, *β*_*xw*_)	(2, 1)
With moderate effect modification (*β*_*w*_, *β*_*xw*_)	(2, 3)
With strong effect modification (*β*_*w*_, *β*_*xw*_)	(1, 5)
With strong effect modification (*β*_*w*_, *β*_*xw*_)	(2, 5)

### Data generation details

The treatment variable is the covariate of primary interest in the IPD-MA; the effect modifier changes the effect of treatment on the outcome when present.

#### The number of studies and subjects

The number of studies in each dataset was given by *N* and was set to 15 or 30. The number of the subjects within each study (*n*_*i*_,*i*=1,...,*N*) followed a log-normal distribution, $\text {LN}(\sigma ^{2}_{lg}=1.5^{2},\kappa =10)$, that was truncated at 20 and 2000 (to avoid very small and large studies), and rounded to the nearest integer value.

#### Treatment status

The prevalence of treatment for each study was generated from a uniform distribution, $p_{x_{i}}\sim U(\theta _{\text {lower}}=0.4,\theta _{\text {upper}}=0.6)$. Using these study specific treatment prevalences, we generated *n*_*i*_ random variables from a Bernoulli distribution for each subject, as $\phantom {\dot {i}\!}x_{ij}\sim \text {Bernoulli}(p_{x_{i}}).$ These *x*_*ij*_ represented the treatment status of subject *j* in study *i*.

#### Effect modifier

We generated a binary effect modifier. First, *N* study-specific effect modifier prevalences were generated as $p_{w_{i}}\sim \text {Uniform}(\theta _{w_{\text {lower}}}=0.1,\theta _{w_{\text {upper}}}=0.9)$. Then, using these probabilities, we obtained the effect modifiers from the Bernoulli distributions, $\phantom {\dot {i}\!}w_{ij}\sim \text {Bernoulli}(p_{w_{i}})$.

#### Outcomes

We generated the outcome *y*_*ij*_ based on the generated values of the treatment and effect modifier, as well as the regression coefficients that described the association of each of these with the binary outcome, using the following equation:

10 $$ \text{logit}(p_{ij})=\beta_{0}+\mu_{0,i}+(\beta_{1}+\mu_{1,i})x_{ij}+\beta_{w}w_{ij}+\beta_{xw}x_{ij}w_{ij}.  $$

*β*_0_, the fixed intercept, was set based on the given value of prevalence *p*_pre_, where $\beta _{0}=\text {log}\left (\frac {p_{\text {pre}}}{1-p_{\text {pre}}}\right)$. The prevalence (*p*_pre_) was set at 0.3 or 0.7. The random intercepts for individuals within each study were $\mu _{0,i}\sim \text {Normal}(0,\sigma ^{2}_{\mu _{0}})$, where $\sigma ^{2}_{\mu _{0}}$ was given. The true pooled treatment effect was *β*_1_. Furthermore, *μ*_1,*i*_ was the study-specific random effect for the slope, which followed a normal distribution with zero mean and variance *τ* ^2^. *β*_*w*_ and (*β*_*w*_+*β*_*xw*_) were the log odds ratio of the effect modifier in untreated and treated individuals, respectively. The parameter value used to generate the random intercepts ($\sigma ^{2}_{\mu _{0}}$) was given by 1 and the fixed interested *β*_1_ was given by log(1.3). The parameter used to generate the random slopes (*τ* ^2^) was set to 0.5, 1, or 1.5.

Using Eq. 10, we obtained *p*_*ij*_. Participant level probabilities of outcome were calculated as $\pi _{ij}=\frac {e^{p_{ij}}}{1-e^{p_{ij}}}$, then *y*_*ij*_ was generated from a Bernoulli(*π*_*ij*_) distribution.

### Datasets

We contemplated two scenarios, including (i) no effect modification and (ii) effect modification, by varying the data generation parameters (*β*_*w*_,*β*_*xw*_).

Our rationale was to evaluate each measure of heterogeneity according to the following:

- **(i)** Did the measures of heterogeneity increase with increasing *τ* ^2^ in datasets that were generated such that there was no effect modification?
- **(ii)** Did the measures of heterogeneity decrease when the effect modifier and an interaction term between treatment and the effect modifier were included in the model when effect modification was present?

Furthermore, we investigate whether the simulation-based *I* ^2^ satisfied the criteria proposed by Higgins et al.: (i) monotonically increasing with increasing between-study variance; (ii) not varied by changing scale; and (iii) not affected by the number of studies [5].

#### IPD-MA with no effect modification

To generate datasets with no effect modification, we set *β*_*w*_ and *β*_*xw*_ to zero.

#### IDP-MA with effect modification

We varied *β*_*w*_ and *β*_*xw*_ to generate datasets with weak or strong effect modification, as presented in Table 1.

### Data analysis

For each generated dataset, we considered both two-stage and one-stage approaches to quantifying heterogeneity.

#### Two-stage approach

In this approach, each study is analyzed separately then pooled together using methods described in “Between-study variance (*τ*^2^)” section [12].

In the first stage, we considered two logistic regression models for each study in the dataset: (i) a crude model (logit(*p*_*j*_)=*β*_0_+*β*_1_ *x*_*j*_) and (ii) an effect modification model (logit(*p*_*j*_)=*β*_0_+*β*_1_ *x*_*j*_+*β*_2_ *w*_*j*_+*β*_3_ *x*_*j*_ *w*_*j*_), where *p*_*j*_ was the true response probability for the positive result of the *j*th individual in this study, *β*_0_ represented the intercept, *x*_*j*_ indicated the treatment status, and *w*_*j*_ was the effect modifier for the *j*th individual in this study.

When the IPD were generated without effect modification, we fitted the crude model to estimate the pooled treatment effect. When the IPD were generated with effect modification, we considered a crude model and a model that included the effect modifier, the treatment, and an interaction term between the effect modifier and the treatment to estimate the pooled treatment effect.

In the first stage, we estimated the log odds ratio $\hat {\beta }_{1i}$ (*i*=1,...,*N*) from each study. In the second stage, we pooled these together via the DerSimonian and Laird method and, estimated the between-study variance ($\hat {\tau }^{2}$) and the pooled treatment effect. We also applied the methods described in “*I*^2^ statistic” and “*R* statistic” sections to estimate the $I^{2}_{\mathrm {two-stage}}$ and $R^{2}_{\mathrm {two-stage}}$ for quantifying the heterogeneity in a two-stage IPD-MA.

#### One-stage approach

For each generated dataset, we fitted a logistic regression with random intercept and slope for studies, estimated via adaptive Gauss-Hermite quadrature. Similar to the two-stage approach, we considered the following models: (i) a crude model (logit(*p*_*ij*_)=(*β*_0_+*μ*_0*i*_)+(*β*_1_+*μ*_1*i*_)*x*_*ij*_) and (ii) an effect modification model (logit(*p*_*ij*_)=(*β*_0_+*μ*_0*i*_)+(*β*_1_+*μ*_1*i*_)*x*_*ij*_+*β*_2_ *w*_*ij*_+*β*_3_ *x*_*ij*_ *w*_*ij*_).

In all models, *p*_*ij*_ was the true response probability of disease for the *j*th individual in the *i*th study, *x*_*ij*_ indicated treatment status, and *w*_*ij*_ represented the effect modifier. The random intercept and slope were *μ*_0*i*_,*μ*_1*i*_, such that:

$$ \left[\begin{array}{l} \mu_{0i} \\ \mu_{1i} \end{array}\right] \sim \text{MVN} \left(\left[\begin{array}{l} 0 \\ 0 \end{array}\right], \left[\begin{array}{ll} \tau^{2}_{0}&\rho\tau_{0}\tau_{1} \\ \rho\tau_{0}\tau_{1}&\tau^{2}_{1} \end{array}\right]\right), $$

where $\hat {\tau }_{1}$ was the estimated between-study variance of the treatment effect. We estimated $I^{2}_{\mathrm {one-stage}}$ via the simulation-based method, and $R^{2}_{\mathrm {one-stage}}$ was computed by the ratio of the estimated variance of *β*_1_ under a random slopes model and a fixed slope model with random intercepts, as described in the “*I*^2^ statistic” and “*R* statistic” sections.

### Metrics and performance

We collected *I* ^2^, *R* ^2^, and *τ* ^2^ as estimated from both two-stage and one-stage approaches in each generated dataset for all combinations of data generation parameters. We estimated the median and interquartile range (IQR) from 1000 datasets. If the dataset was generated with effect modification, then the median and IQR of the ratio of the *I* ^2^ as estimated from a crude model to that estimated from a model that included the effect modifier and the interaction between the effect modifier and treatment status $\left (\frac {I^{2}_{\text {emod}}}{I^{2}_{\text {crude}}}\right)$ was collected. Similar measures were reported for *R* ^2^ and *τ* ^2^. We collected the ratios because we wanted to investigate the differences in $\hat {I}^{2}$, $\hat {R}^{2}$, and $\hat {\tau }^{2}$ before and after taking effect modification into account. All statistical analysis were carried out in *R*, version 3.2.3 [23].

## Results

### With no effect modification

**Fig. 1:**
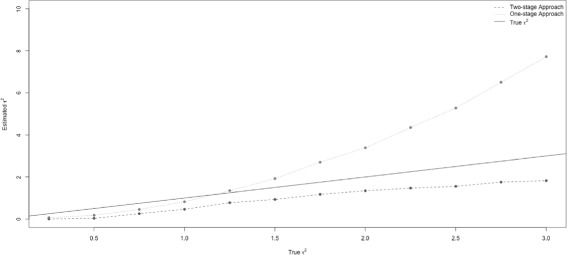
True *τ* ^2^ versus estimated *τ* ^2^. The estimated between-study variances from a conventional two-stage model and a simulation-based one-stage mode are compared with the true between-study variance

Figure 1 shows the estimated between-study variance $\hat {\tau }^{2}$ from both the two-stage (dashed line) and the one-stage (dotted line) approaches versus the true between-study variance, *τ* ^2^ (solid line). As the true *τ* ^2^ increased, the estimated $\hat {\tau }^{2}$ from both approaches also increased. Compared with the estimated $\hat {\tau }^{2}$ from a two-stage model, the $\hat {\tau }^{2}$ from the one-stage model increased more rapidly. The two-stage model always underestimated $\hat {\tau }^{2}$. On the other hand, the one-stage approach very slightly underestimated $\hat {\tau }^{2}$ when the true *τ* ^2^ was small, and it overestimated $\hat {\tau }^{2}$ when the true *τ* ^2^ was larger than 1.3.

**Fig. 2:**
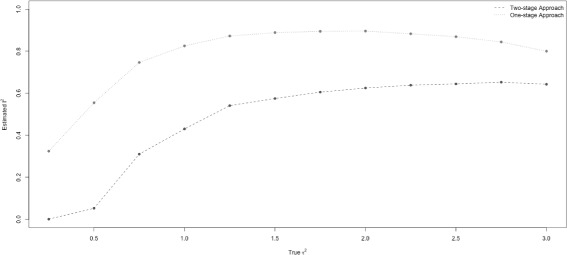
True *τ* ^2^ versus estimated *I* ^2^. The estimated *I* ^2^ from a conventional two-stage model and a simulation-based one-stage model are compared with the true between-study variance. The dashed line and dotted line represented the estimated *I* ^2^ from the two-stage and one-stage models based on its median value across 1000 datasets

Figure 2 shows the conventional *I* ^2^ from the two-stage model (dashed line) and the simulation-based *I* ^2^ from one-stage model (dotted line) versus the true between-study variance *τ* ^2^. Both measures increased, then leveled off as the true between-study variance increased.

Table 2 presents the median value and IQR of the $\hat {I}^{2}$ and $\hat {R}^{2}$ across 1000 datasets from the two-stage and one-stage models for different combinations of data generation parameter values. The median estimated *I* ^2^ and *R* ^2^ from both the two-stage and one-stage model increased as the true between-study variance increased. $\hat {I}^{2}_{\mathrm {two-stage}}$ and $\hat {I}^{2}_{\mathrm {one-stage}}$ were very similar for *N*=15 and *N*=30. However, the *R* ^2^ statistic from both approaches slightly increased as the number of studies increased. Varying the prevalence from 30 to 70% did not affect the estimates of *I* ^2^ and *R* ^2^ via two- and one-stage models.

**Table 2:** Median (IQR) of heterogeneity metrics for the treatment effect when no effect modification was present^a^

*τ* ^2^	Prevalence (%)	Number of studies	$I^{2}_{\mathrm {two-stage}}$	$R^{2}_{\mathrm {two-stage}}$	$I^{2}_{\mathrm {one-stage}}$	$R^{2}_{\mathrm {two-stage}}$
0.5	30	15	0.10 (0.33)	1.22 (0.80)	0.58 (0.41)	1.75 (1.13)
1.0	30	15	0.44 (0.36)	2.30 (1.76)	0.83 (0.15)	3.29 (2.41)
1.5	30	15	0.58 (0.30)	3.22 (2.53)	0.89 (0.09)	5.84 (4.24)
0.5	70	15	0.01 (0.26)	1.00 (0.65)	0.52 (0.47)	1.76 (1.11)
1.0	70	15	0.39 (0.42)	2.12 (1.69)	0.80 (0.18)	3.33 (2.26)
1.5	70	15	0.55 (0.32)	3.07 (2.67)	0.87 (0.11)	5.66 (4.36)
0.5	30	30	0.12 (0.28)	1.29 (0.72)	0.60 (0.32)	1.79 (0.84)
1.0	30	30	0.47 (0.25)	2.44 (1.29)	0.84 (0.09)	3.49 (1.61)
1.5	30	30	0.62 (0.19)	3.54 (1.98)	0.90 (0.05)	6.32 (3.26)
0.5	70	30	0.06 (0.22)	1.14 (0.57)	0.56 (0.34)	1.81 (0.82)
1.0	70	30	0.42 (0.28)	2.30 (1.30)	0.82 (0.11)	3.52 (1.80)
1.5	70	30	0.59 (0.20)	3.37 (1.93)	0.88 (0.07)	6.36 (3.14)

^a^Please note that *I* ^2^ is presented here as a proportion varying from 0 to 1, rather than as a percentage

Furthermore, $\hat {\tau }^{2}$ from the two- and one-stage approaches were similar for different prevalence and number of studies (Additional file 1: Table S1).

### With effect modification

Table 3 presents the median value and IQR of the ratio of *I* ^2^ and *R* ^2^ from a model that ignored the effect modifier to one that included the effect modifier and an interaction term between it and the treatment status across 1000 datasets from the two-stage and one-stage approaches with prevalence =30*%*. Any measure of heterogeneity should be sensitive to changes in heterogeneity. If we did not account for effect modification when it existed, then heterogeneity might arise due to this effect modification [24]. Hence, if the ratio estimators reported in the Table 3 are less than 1, they indicate good sensitivity of the measure to changing heterogeneity.

**Table 3:** Sensitivity of heterogeneity measures to accounting for effect modification when prevalence of the outcome was 30%

			Two-stage approach		One-stage approach	
*τ* ^2^	Number of studies	Strength of effect modification^a^	$\frac {I^{2}_{\text {emod}}}{I^{2}_{\text {crude}}}$	$\frac {R^{2}_{\text {emod}}}{R^{2}_{\text {crude}}}$	$\frac {I^{2}_{\text {emod}}}{I^{2}_{\text {crude}}}$	$\frac {R^{2}_{\text {emod}}}{R^{2}_{\text {crude}}}$
0.5	15	Weak	0.17 (1.00)	0.89 (0.38)	1.00 (0.41)	0.91 (0.25)
1.0	15	Weak	0.01 (0.57)	0.60 (0.34)	0.99 (0.07)	0.81 (0.25)
1.5	15	Weak	0.06 (0.59)	0.50 (0.30)	0.98 (0.05)	0.75 (0.18)
0.5	15	Moderate	0.02 (1.00)	0.86 (0.39)	0.56 (0.68)	0.86 (0.34)
1.0	15	Moderate	0.08 (0.87)	0.68 (0.46)	0.82 (0.28)	0.98 (0.40)
1.5	15	Moderate	0.34 (0.77)	0.65 (0.39)	0.82 (0.25)	1.06 (0.46)
0.5	15	Strong	0.01 (1.00)	0.82 (0.42)	0.11 (0.23)	0.90 (0.37)
1.0	15	Strong	0.32 (1.00)	0.81 (0.43)	0.20 (0.28)	1.07 (0.57)
1.5	15	Strong	0.42 (0.93)	0.75 (0.46)	0.22 (0.29)	1.31 (0.86)
0.5	30	Weak	0.01 (1.00)	0.78 (0.39)	1.00 (0.27)	0.88 (0.19)
1.0	30	Weak	0.01 (0.36)	0.53 (0.22)	1.00 (0.04)	0.78 (0.16)
1.5	30	Weak	0.16 (0.49)	0.47 (0.22)	0.98 (0.03)	0.74 (0.14)
0.5	30	Moderate	0.01 (1.00)	0.77 (0.40)	0.59 (0.46)	0.86 (0.29)
1.0	30	Moderate	0.01 (0.54)	0.65 (0.30)	0.82 (0.19)	0.99 (0.33)
1.5	30	Moderate	0.19 (0.61)	0.59 (0.30)	0.82 (0.17)	1.07 (0.31)
0.5	30	Strong	0.01 (1.00)	0.79 (0.37)	0.09 (0.15)	0.95 (0.37)
1.0	30	Strong	0.01 (0.68)	0.70 (0.34)	0.16 (0.17)	1.10 (0.46)
1.5	30	Strong	0.33 (0.73)	0.71 (0.33)	0.16 (0.17)	1.23 (0.69)

Median (IQR) was presented

We present the ratios of the measure estimated from a model that ignored the effect modifier to one that included the effect modifier and an interaction term between it and the treatment status

^a^Effect modification was classified as weak when *β*_*w*_=1, *β*_*xw*_=1, as moderate when *β*_*w*_=1, *β*_*xw*_=3, and as strong when *β*_*w*_=2, *β*_*xw*_=5

When the strength of effect modification was weak, the ratio estimators for $I^{2}_{\mathrm {two-stage}}$ were well below 1, while the ratio estimators for $I^{2}_{\mathrm {one-stage}}$ were close to 1. When the strength of effect modification was moderate or strong, we found the ratio estimators for $I^{2}_{\mathrm {one-stage}}$ were below 1, suggesting the estimated $I^{2}_{\mathrm {one-stage}}$ was sensitive to changing heterogeneity. When the prevalence increased from 30 to 70%, in the two-stage model, almost all values of the ratio estimator for *τ* ^2^ were equal to 1 (Additional file 1: Table S3), as were most values of the ratio estimator for $I^{2}_{\mathrm {two-stage}}$ (Additional file 1: Table S2). The estimated *R* ^2^ from the two-stage model had similar performance. Conversely, most of the ratio estimators for $I^{2}_{\mathrm {one-stage}}$ were less than 1 when we fixed the prevalence to be 70% and varied other parameter values. However, the convergence rate for one-stage approach decreased as the strength of effect modification became stronger (data not shown).

When the number of studies and prevalence were 30 and 30%, most of ratio estimators for $I^{2}_{\mathrm {two-stage}}$ were equal to 0.01. This occurred because the estimated $\tau ^{2}_{\mathrm {two-stage}}$ from the effect modification model was close to zero (Additional file 1: Table S3).

Furthermore, in Additional file 1: Table S3, the ratio estimators for *τ* ^2^ in the two-stage model were all less than or equal to 1. However, most of ratio estimators for *τ* ^2^ in the one-stage model were larger than 1.

## Discussion

IPD-MA are the gold standard of meta-analytic approaches. While the primary objective of most IPD-MA is to estimate pooled treatment effects, quantifying inter-study heterogeneity of those effects is also an important goal. Most statisticians agree that a one-stage approach is the best and most flexible approach to use when analyzing data from IPD-MA. However, how best to quantify inter-study heterogeneity in that case is unclear [3, 5, 12], and most IPD-MA of binary outcomes do not report any measure of heterogeneity [6].

In this work, we considered using usual measures of heterogeneity based on two-stage approaches, as well as novel approaches based on a one-stage model. We evaluated both two-stage and one-stage approaches via simulation studies. In the two-stage approach, we used the usual *I* ^2^ and *R* ^2^ statistics proposed by Higgins et al. to measure heterogeneity [5]. In the one-stage approach, we adapted a simulation-based ICC proposed by Goldstein et al. to estimate the *I* ^2^, as well as considering the *R* ^2^ based on the one-stage model.

Our results demonstrated that when there was no effect modification, the estimated *τ* ^2^ from the two-stage model was always underestimated. When using a one-stage approach, the estimated *τ* ^2^ was underestimated when the true *τ* ^2^ was small, but overestimated when the true *τ* ^2^ was large. Correspondingly, we may assume that the estimated *I* ^2^ from the two-stage model was underestimated, whereas the simulation-based *I* ^2^ in the one-stage model was underestimated when inter-study heterogeneity was small and overestimated when it was large. Both the two-stage *I* ^2^ and one-stage *I* ^2^ increased as the true *τ* ^2^ increased.

Including a variable and the interaction of that variable and the treatment of interest when effect modification is present should decrease the estimated between-study heterogeneity. In the presence of weak effect modification, the estimated *I* ^2^ from the two-stage model that accounted for the effect modification was less than that from a model that did not. Nevertheless, the estimated *I* ^2^ from the one-stage approach that accounted for effect modification quantified heterogeneity well when the strength of effect modification was moderate or strong. The *I* ^2^ from the two-stage model was less sensitive to reflect the strength of effect modification when the number of studies was large and prevalence was low. Overall, this suggests that using the simulation-based *I* ^2^ based on one-stage model is preferable.

Differences between measures of heterogeneity in the two-stage and one-stage approaches might be due to real differences in the methods, or because slightly different models were used. In the one-stage approach, we only considered models that fit a random intercept and slope, while the two-stage approaches fit just a random slope. However, these are the approaches most commonly used [6].

### Strengths of the work

We have proposed a simulation-based *I* ^2^ to use in one-stage IPD-MA of binary outcomes. We have shown that this *I* ^2^ satisfies the conditions proposed by Higgins et al., for any measures quantifying heterogeneity, i.e., (i) the measurement function should monotonically increase with increasing between-study variance *τ* ^2^ and (ii) not be affected by the number of studies *N* [5]. Moreover, we have shown that the simnulation-based *I* ^2^ is sensitive to changes in heterogeneity.

When the outcome is binary, the within-study variance varies across the studies as between-study variance increases [7]. As a result, the assumption of equal estimated sampling variances across all studies, as in Higgins and Thompson’s paper [5], does not hold, and Higgins’s *I* ^2^ may be biased. For that reason, we would expect the simulation-based *I* ^2^ based on the one-stage approach to have better performance than the conventional *I* ^2^ based on the two-stage approach.

Using a heterogeneity measure based on the one-stage model is also advantageous, because the one-stage approach allows investigation of patient- and study-level covariates, and the treatment effect can be adjusted for covariates at the participant- or study-level [18]. Moreover, the one-stage model allows MA of dose-response curves (e.g., allowing non-linearity), improves power for interactions [25, 26], and allows better control of effect modification by patient- and study-level covariates than the two-stage approach [3, 17, 27].

While we investigated its performance for binary outcomes, using the ICC as an *I* ^2^ for continuous outcomes in the context of a mixed model would be possible, though to our knowledge has not been used like this.

### Limitations

There are several limitations in this work. We only considered the ICC estimator proposed by Goldstein to estimate the *I* ^2^ in one-stage IPD-MA of binary outcomes. However, there are several other measures that have been proposed as estimators of the ICC for binary data [14, 21, 22]. Wu et al. discussed five different methods to estimate the ICC with binary outcomes: an analysis of variance (ANOVA) estimator, the Fleiss-Cuzick estimator, the Pearson estimator, an estimator based on generalized estimating equations (GEE), and an estimator from the random intercept logistic model [20]. These could be adapted to estimate *I* ^2^ in one-stage IPD-MA. On the other hand, the measure we have proposed is easy to estimate.

Moreover, GLMMs estimated via adaptive quadrature sometimes do not converge in the one-stage model [19]. Indeed, we observed a sometimes important rate of non-convergence when the strength of effect modification was strong and the prevalence was high. Other estimation approaches such as penalized quasi-likelihood (PQL) or Bayesian multilevel models might be interesting to explore [28, 29]. While convergence of PQL is more likely, estimates can be biased with few subjects per cluster, low event rates, or high inter-cluster variability [7, 29, 30].

For the two-stage approaches, we estimated *τ* ^2^ via the method of moments estimator of DerSimonian and Laird, despite more recent evidence suggesting that the Paule and Mandel estimator is preferred [31].

Finally, we invsestigated a finite number of data generation parameters. In particular, we considered datasets of 15 or 30 studies, whereas it may have been interesting to consider fewer (e.g., 5).

## Conclusion

When a one-stage approach for IDP-MA of binary outcomes is preferred, heterogeneity should be quantified via the model estimated. In that case, we have proposed a simulation-based *I* ^2^ that performs as well or better than the conventional *I* ^2^ based on a two-stage approach. The *R* ^2^ based on the one-stage model also performed adequately but is more difficult to interpret.

## Additional file

Additional file 1
**Table S1.** The Median (IQR) of the estimated *τ* ^2^ when no effect modification was present. **Table S2.** Sensitivity of heterogeneity measures to accounting for effect modification when prevalence of the outcome was 70%. Median (IQR) was presented. **Table S3.** Sensitivity of heterogeneity measures $\left(\frac{\tau_{\text{emod}}^{2}}{\tau^{2}_{\text{crude}}}\right)$ to accounting for effect modification. Median (IQR) was presented. (PDF 145 kb)

## Abbreviations

AD-MA: Aggregate data meta-analysis
ANOVA: Analysis of variance
GEE: Generalized estimating equation
GLMM: Generalized linear mixed model
ICC: Intraclass correlation coefficient
IPD-MA: Individual participant data meta-analysis
IQR: Interquartile range
MA: Meta-analysis
PQL: Penalized quasi-likelihood
