# Supplementary material for: Quantifying heterogeneity in individual participant data meta-analysis with binary outcomes
Source: Syst Rev. 2017 Dec 6;6:243. doi: 10.1186/s13643-017-0630-4 (PMC5718085; doi:10.1186/s13643-017-0630-4)
Supplement: Additional file 1 — Table S1. The Median (IQR) of the estimated τ 2 when no effect modification was present. Table S2. Sensitivity of heterogeneity measures to accounting for effect modification when prevalence of the outcome was 70%. Median (IQR) was presented. Table S3. Sensitivity of heterogeneity measures $\left(\frac{\tau_{\text{emod}}^{2}}{\tau^{2}_{\text{crude}}}\right)$τemod2τcrude2 to accounting for effect modification. Median (IQR) was presented. (PDF 145 kb) [file 13643_2017_630_MOESM1_ESM.pdf]

Table S1: The Median (IQR) of the estimated τ^2 when no effect modification was present.

τ^2	Prevalence	Number of studies	$\tau^2_{two-stage}$	$\tau^2_{one-stage}$
0.5	30%	15	0.06 (0.26)	0.21 (0.35)
1.0	30%	15	0.47 (0.65)	0.84 (0.83)
1.5	30%	15	0.96 (1.08)	2.00 (1.67)
0.5	70%	15	0.01 (0.20)	0.18 (0.35)
1.0	70%	15	0.41 (0.67)	0.84 (0.79)
1.5	70%	15	0.89 (1.01)	1.92 (1.67)
0.5	30%	30	0.07 (0.20)	0.20 (0.26)
1.0	30%	30	0.51 (0.48)	0.86 (0.57)
1.5	30%	30	1.03 (0.73)	2.08 (1.15)
0.5	70%	30	0.04 (0.15)	0.20 (0.26)
1.0	70%	30	0.46 (0.48)	0.89 (0.63)
1.5	70%	30	0.98 (0.71)	2.13 (1.17)

Table S2: Sensitivity of heterogeneity measures[1] to accounting for effect modification when prevalence of the outcome was 70%. Median (IQR) was presented.

			Two-stage approach		One-stage approach	
τ^2	Number of studies	Strength of effect modification[2]	$\frac{I^2_{emod}}{I^2_{crude}}$	$\frac{R^2_{emod}}{R^2_{crude}}$	$\frac{I^2_{emod}}{I^2_{crude}}$	$\frac{R^2_{emod}}{R^2_{crude}}$
0.5	15	Weak	1.00 (0.01)	1.00 (0.01)	0.84 (0.39)	0.83 (0.17)
1.0	15	Weak	0.85 (1.00)	0.94 (0.34)	0.90 (0.18)	0.81 (0.17)
1.5	15	Weak	0.54 (1.00)	0.71 (0.50)	0.90 (0.18)	0.79 (0.16)
0.5	15	Moderate	1.00 (0.01)	1.00 (0.01)	0.31 (0.47)	0.87 (0.19)
1.0	15	Moderate	1.00 (0.66)	1.00 (0.23)	0.41 (0.38)	0.90 (0.23)
1.5	15	Moderate	0.91 (0.96)	0.97 (0.32)	0.44 (0.37)	0.97 (0.31)
0.5	15	Strong	1.00 (0.01)	1.00 (0.01)	0.01 (0.01)	0.89 (0.19)
1.0	15	Strong	1.00 (0.01)	1.00 (0.01)	0.01 (0.15)	0.95 (0.30)
1.5	15	Strong	1.00 (0.12)	1.00 (0.10)	0.06 (0.21)	1.05 (0.49)
0.5	30	Weak	1.00 (0.01)	1.00 (0.01)	0.86 (0.32)	0.84 (0.12)
1.0	30	Weak	0.34 (1.00)	0.78 (0.39)	0.90 (0.12)	0.80 (0.10)
1.5	30	Weak	0.24 (0.71)	0.62 (0.33)	0.89 (0.12)	0.78 (0.11)
0.5	30	Moderate	1.00 (0.01)	1.00 (0.01)	0.28 (0.31)	0.87 (0.16)
1.0	30	Moderate	1.00 (1.00)	1.00 (0.24)	0.40 (0.25)	0.92 (0.19)
1.5	30	Moderate	0.69 (1.00)	0.85 (0.35)	0.43 (0.28)	1.00 (0.22)
0.5	30	Strong	1.00 (0.01)	1.00 (0.01)	0.01 (0.05)	0.88 (0.16)
1.0	30	Strong	1.00 (0.05)	1.00 (0.02)	0.05 (0.13)	0.96 (0.19)
1.5	30	Strong	1.00 (0.35)	1.00 (0.20)	0.06 (0.11)	1.09 (0.32)

[1]We present the ratios of the measure estimated from a model that ignored the effect modifier to one that included the effect modifier and an interaction term between it and treatment status.

[2]Effect modification was classified as weak when $\beta_w = 1$, $\beta_{xw} = 1$, as moderate when $\beta_w = 1$, $\beta_{xw} = 3$, and as strong when $\beta_w = 2$, $\beta_{xw} = 5$.

Table S3: Sensitivity of heterogeneity measures[3] ($\frac{\tau^2_{emod}}{\tau^2_{crude}}$) to accounting for effect modification. Median (IQR) was presented.

			Two-stage approach		One-stage approach	
τ^2	Number of studies	Strength of confounding[4]	$\frac{\tau^2_{emod}}{\tau^2_{crude}}$ $(pre = 30\%)$	$\frac{\tau^2_{emod}}{\tau^2_{crude}}$ $(pre = 70\%)$	$\frac{\tau^2_{emod}}{\tau^2_{crude}}$ $(pre = 30\%)$	$\frac{\tau^2_{emod}}{\tau^2_{crude}}$ $(pre = 70\%)$
0.5	15	Weak	0.21 (1.00)	1.00 (0.01)	1.35 (1.55)	1.21 (1.12)
1.0	15	Weak	0.01 (0.85)	1.00 (1.00)	1.44 (0.36)	1.30 (0.59)
1.5	15	Weak	0.06 (0.78)	0.64 (1.00)	1.41 (0.38)	1.32 (0.36)
0.5	15	Moderate	0.03 (1.00)	1.00 (0.01)	0.93 (2.58)	1.19 (2.38)
1.0	15	Moderate	0.11 (1.00)	1.00 (0.57)	2.16 (2.43)	1.60 (1.39)
1.5	15	Moderate	0.49 (1.05)	1.00 (0.94)	2.47 (1.61)	1.79 (1.02)
0.5	15	Strong	0.01 (1.00)	1.00 (0.01)	1.28 (2.67)	1.35 (2.40)
1.0	15	Strong	0.56 (1.00)	1.00 (0.01)	2.89 (4.30)	1.95 (2.43)
1.5	15	Strong	0.74 (1.48)	1.00 (0.29)	3.87 (4.23)	2.09 (1.75)
0.5	30	Weak	0.01 (1.00)	1.00 (0.01)	1.38 (1.02)	1.28 (0.93)
1.0	30	Weak	0.01 (1.00)	0.43 (1.00)	1.42 (0.39)	1.31 (0.37)
1.5	30	Weak	0.19 (0.62)	0.28 (0.94)	1.41 (0.25)	1.33 (0.26)
0.5	30	Moderate	0.01 (1.00)	1.00 (0.01)	1.12 (1.69)	1.32 (1.62)
1.0	30	Moderate	0.01 (0.98)	1.00 (1.00)	2.16 (1.50)	1.70 (0.87)
1.5	30	Moderate	0.26 (1.00)	0.98 (1.07)	2.47 (1.09)	1.86 (0.66)
0.5	30	Strong	0.01 (1.00)	1.00 (0.01)	1.50 (2.21)	1.39 (2.19)
1.0	30	Strong	0.01 (1.00)	1.00 (0.01)	2.94 (2.66)	1.83 (1.23)
1.5	30	Strong	0.59 (1.41)	1.00 (0.76)	3.58 (2.33)	2.13 (1.03)

[3]We present the ratios of the measure estimated from a model that ignored the effect modifier to one that included the effect modifier and an interaction term between it and treatment status.

[4]Effect modification was classified as weak when $\beta_w = 1$, $\beta_{xw} = 1$, as moderate when $\beta_w = 1$, $\beta_{xw} = 3$, and as strong when $\beta_w = 2$, $\beta_{xw} = 5$.
